# Emerging trends and hot spots in subacute thyroiditis research from 2001 to 2022: A bibliometric analysis

**DOI:** 10.3389/fendo.2023.1144465

**Published:** 2023-03-17

**Authors:** Cheng Xu, Rui Jiang, Jiang-yu Liu

**Affiliations:** The First Clinical Medical College of Nanjing University of Chinese Medicine, Nanjing, China

**Keywords:** subacute thyroiditis (SAT), bibliometric analysis, CiteSpace, VOSviewer, hot spots

## Abstract

**Background:**

Subacute thyroiditis (SAT) is the most prevalent self-limiting thyroid disease that causes pain, accounting for about 5% of all clinical thyroid disorders. Numerous clinically noteworthy results have been published in this area over the last 20 years. However, no article has comprehensively assessed the relevant literature yet. We conducted a bibliometric analysis of SAT to provide light on the dynamic nature of scientific advancement and aid researchers in gaining a global perspective while examining research core themes and hotspots.

**Methods:**

SAT-related articles and reviews from 2001 to 2022 were retrieved from the Science Citation Index-Expanded of Web of Science Core Collection (WoSCC). We analyzed current research trends and hotspots in this area using CiteSpace and Vosviewer.

**Results:**

A total of 568 studies associated with SAT research were published in 282 academic journals by 2,473 authors in 900 institutions from 61 countries/regions. The United States was a crucial link in inter-country/region collaboration and was the most frequently involved country in international cooperation. The University of Missouri System was the top organization, and Braley-Mullen H. was the most productive researcher. *Thyroid* published the most papers, with 36 publications. The most co-cited article was “Clinical features and outcome of subacute thyroiditis in an incidence cohort: Olmsted County, Minnesota, study” (by Fatourechi V., 2003). The clustered network and timeline view of keywords showed that the prevalence, diagnosis, and treatment of SAT were the research core themes during the past 20 years. Analysis of keyword bursts indicated that the clinical characteristic and the influence of COVID-19 on SAT appeared to be the current research hotspots.

**Conclusion:**

This bibliometric analysis conducted a thorough review of the SAT research. The clinical characteristics and the genetic background of SAT under the influence of COVID-19 are current research hotspots. However, there is still a need for further study and global collaboration. Our findings can aid researchers in understanding the current status of SAT research and immediately pinpoint new directions for further investigation.

## 1 Introduction

Subacute thyroiditis (SAT) is the most prevalent self-limiting thyroid disease that causes pain, accounting for about 5% of all clinical thyroid disorders (1). Most patients are middle-aged, and women are four to seven times more likely than males to have the condition (2, 3). SAT manifests as anterior neck pain and tenderness during the physical examination with various systemic symptoms, including fever, chills, palpitation, weight loss, and malaise (4), typically characterized by three clinical processes: thyrotoxicosis, hypothyroidism, and return to normal thyroid function. Since SAT is a self-limiting disease, nonsteroidal anti-inflammatory medications (NSAIDs) and corticosteroids are suggested therapies to treat the signs and symptoms and lessen inflammation (5, 6). However, even among properly treated patients, the recurrence rate for SAT is relatively high, varying from 1.6% to more than 20%, which is associated with the presence of specific types of human leukocyte antigens (HLA) (7). High recurrence rates and prolonged treatment time have become severe problems in treating SAT (8).

Although the cause and pathogenesis of SAT have long been unknown, viral infection or allergic reaction following viral infection are frequently implicated. The onset of SAT is related to several viruses, including the Coxsackie virus, Echovirus, influenza virus, parvovirus B19, mumps, rubella virus, HIV, Epstein-Barr virus, hepatitis E, measles, and dengue virus (9, 10). Since the winter of 2019, the novel coronavirus (COVID-19) outbreak has spread globally. As the pandemic spreads, there is more and more proof that SAT and SARS-CoV-2 infection are related. Numerous SAT cases involving COVID-19 infection or vaccination have already been published (11–13). However, it remains an open question whether SAT is a complication of COVID-19 or a side effect of vaccination based on available research data.

Over the past two decades, there have been many published research findings on SAT. New methods for reviewing and analyzing trends are required because of the literature’s increasing growth. Bibliometrics is a method for comprehensively assessing a research field that measures scientific data distribution, traits, and regulations from various angles, displaying the field’s macro knowledge structure and development trend (14). No bibliometric study has been reported to evaluate the associated literature comprehensively, summarize the latest trend, and predict research hotspots of SAT. Our study aims to analyze research core themes, explore the frontier issues of SAT from 2001-2022 and provide a comprehensive perspective and guidance for other researchers.

## 2 Methods

### 2.1 Data sources and search strategies

**Figure 1:**
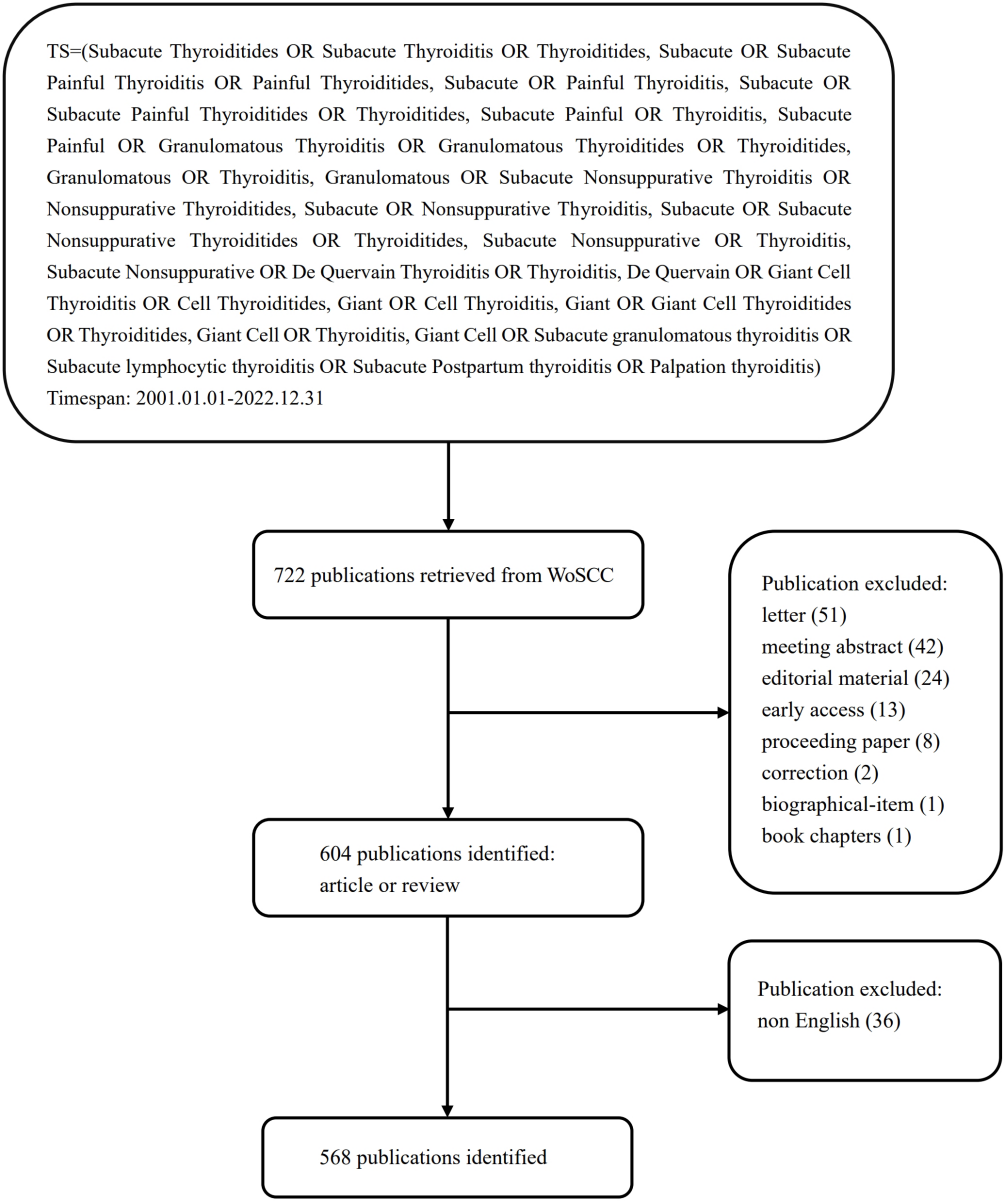
Flowchart for including and excluding literature studies.

We thoroughly searched the Science Citation Index-Expanded (SCI-E) of Web of Science Core Collection (WoSCC) database for the period 2001–2022. All searches were finished and independently verified by two authors on December 31, 2022, in order to remove the bias introduced by daily database changes. The selection criteria were as follows: (1) timespan: 2001.01.01–2022.12.31; (2) document type: article or review; (3) language: English. The details of the search strategy are presented in **Figure 1**.

### 2.2 Bibliometric analysis

For further analysis and to describe all aspects of the literature about SAT research, we converted all WoSCC data that complied with requirements to TXT format and imported it into CiteSpace (V6.1 R6 Basic) and VOSviewer (V1.6.18). CiteSpace is a freely available program written in Java for visualizing and examining trends and patterns in scientific publications (14). The version of this software is constantly updated, and the latest version V6.1R6 Basic was used in this study. CiteSpace was utilized in this study to analyze and display data on the annual growth patterns of publication outputs, countries/regions, institutions, authors, and co-cited authors, the occurrence of keywords, and reference burst. VOSviewer, a Java-based bibliometric mapping program created by Leiden University, excels in handling sizable bibliometric maps based on network data and displaying scientific knowledge (15). Vosviewer was used to study and visualize the analysis of co-cited references, journals, and co-cited journals.

## 3 Results

### 3.1 Annual growth trend of publications

**Figure 2:**
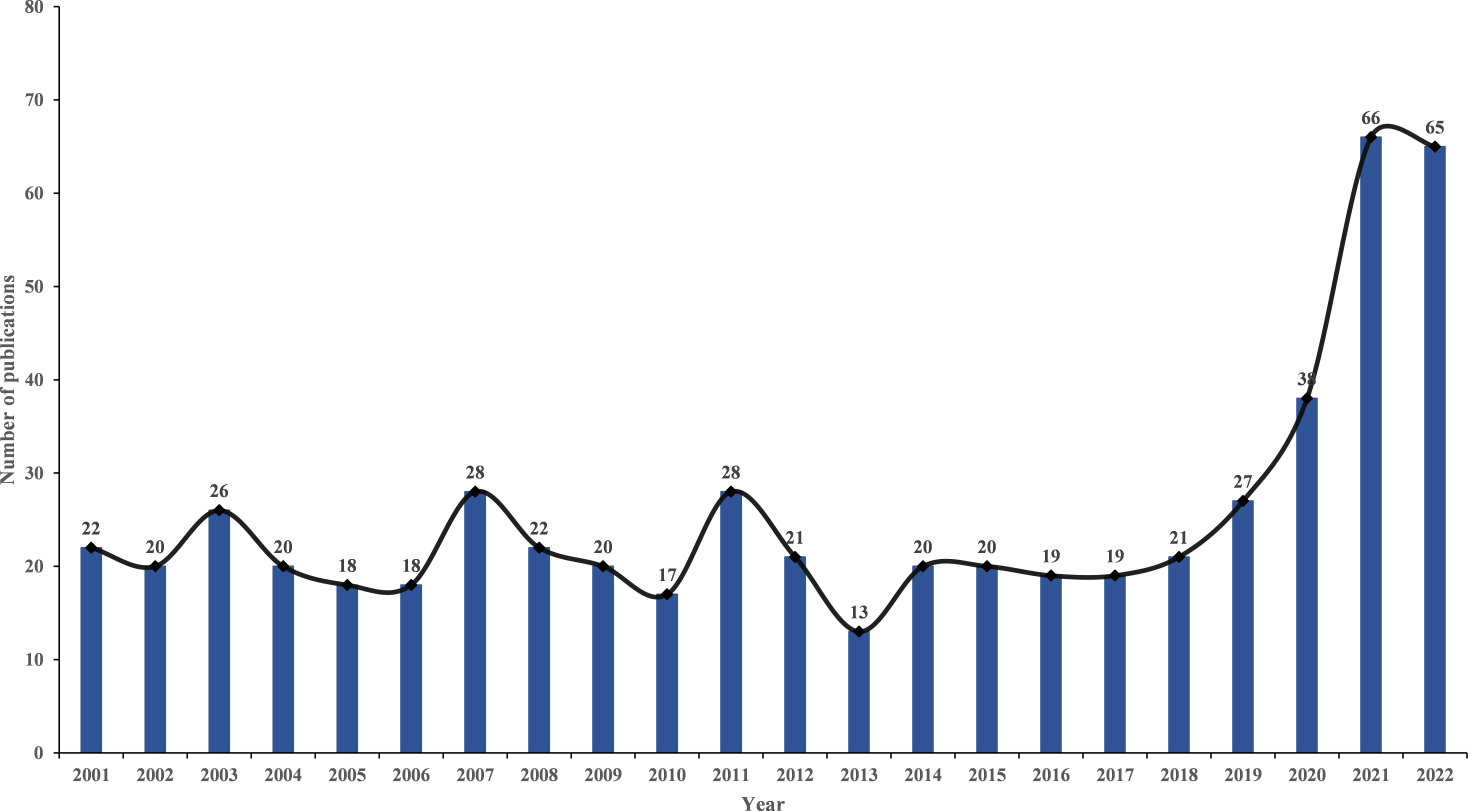
Trend of publications about the research of SAT.

Following the criteria for data selection, 568 SAT studies were found in WoSCC between 2001 and 2022, including 468 original articles (82.4%) and 100 reviews (17.6%). **Figure 2** illustrates that from 2001 to 2018, the volume of publications relating to SAT research tended to be steady. However, there has been an increasing trend in the number of publications since 2019.

### 3.2 Analysis of country/region and institutions

**Figure 3:**
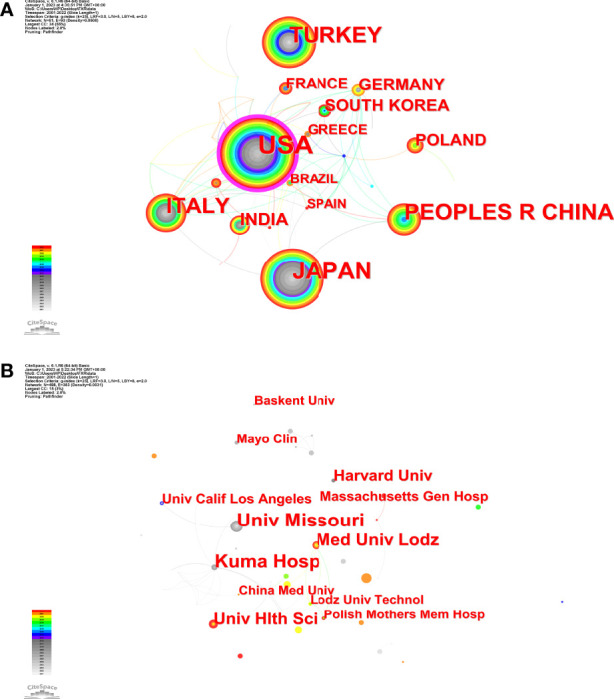
The network maps **(A)** Countries; **(B)** Institutions. The size of node represents the co-occurrence frequencies while the links reflect the co-occurrence relationships. The color of node and line indicates different years.

Between 2001 and 2022, 568 studies were co-authored by 900 institutions across 61 nations and regions. United States (n = 126, 22.18%) placed first in terms of publications, followed by Japan (n = 75, 13.20%), Turkey (n = 64, 11.44%), China (n = 63, 11.09%), and Italy (n = 52, 9.16%). Moreover, The United States has the highest centrality (0.36) (**Figure 3A**), indicating its importance as a link in international and regional cooperation (16). As for the institutions, first place went to the University of Missouri System (n = 19, 3.35%), followed by Kuma Hospital (n = 16, 2.82%), University of Pisa (n = 15, 2.64%), University of California System (n =14, 2.47%), and Harvard University (n = 11, 1.94%). However, network density was only 0.0031(**Figure 3B**), indicating that institutions did not cooperate well enough (16).

### 3.3 Analysis of journals and co-cited journals

**Figure 4:**
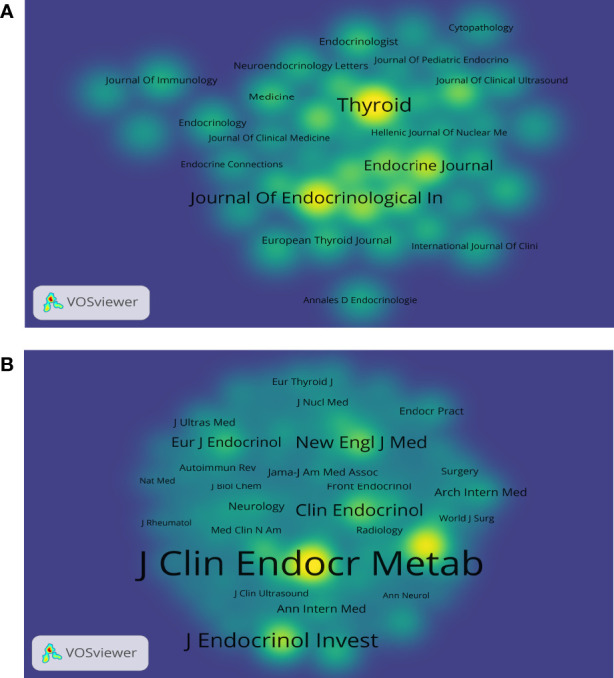
The density maps **(A)** Journals; **(B)** Co-cited journals. The size of the word and round, and the opacity of yellow are positively associated with the cited frequency **(A)**. The size of the word and round, and the opacity of yellow are positively associated with the co-cited frequency **(B)**.

The 568 SAT research publications were printed in 281 academic journals. **Table 1** lists the top 10 journals by productivity and co-citations. *Thyroid* (n = 36, 6.34%), which had an IF of 6.506 in 2022, published the most studies in this field, followed by *Journal of Endocrinological Investigation* (n = 26, 4.58%), *Endocrine Journal* (n = 18, 3.17%), *Journal of Clinical Endocrinology & Metabolism* (n = 14, 2.47%), and *Endocrine* (n = 12, 2.11%). There were three journals in the Q1 JCR (Journal Citation Reports) division, and *Thyroid* had the highest impact factor (IF) (IF = 6.506). The top 50 journals with the highest overall link strength out of all the included journals were chosen to create the density map, which clearly shows the productive journals (**Figure 4A**). As for the most frequently co-cited journals in **Table 1**, *Journal of Clinical Endocrinology & Metabolism* (n = 1350) ranked first, followed by *Thyroid* (n = 980), *Journal of Endocrinological Investigation* (n = 578), *New England Journal of Medicine* (n = 387), and *Clinical Endocrinology* (n= 382). Seven journals were also found in the Q1 JCR division, with Lancet having the highest IF (IF = 202.731). The density map in **Figure 4B** shows the top 50 co-cited journals selected among the publications with the highest overall link strength.

**Table 1:** Top 10 productive journals and co-cited journals of SAT research.

Rank	Productive Journal	Count	IF	JCR(2022)	Co-cited journal	Citation	IF	JCR(2022)
1	Thyroid	36	6.506	Q1	Journal of Clinical Endocrinology & Metabolism	1350	6.134	Q1
2	Journal Of Endocrinological Investigation	26	5.467	Q2	Thyroid	980	6.506	Q1
3	Endocrine Journal	18	2.86	Q4	Journal of Endocrinological Investigation	578	5.467	Q2
4	Journal of Clinical Endocrinology & Metabolism	14	6.134	Q1	New England Journal of Medicine	387	176.079	Q1
5	Endocrine	12	3.925	Q3	Clinical Endocrinology	382	3.523	Q3
6	Clinical Endocrinology	11	3.523	Q3	Lancet	240	202.731	Q1
7	Diagnostic Cytopathology	11	1.39	Q4	Endocrine Journal	236	2.86	Q4
8	Frontiers In Endocrinology	10	6.055	Q1	European Journal of Endocrinology	227	6.558	Q1
9	Internal Medicine	9	1.282	Q4	Internal Medicine	190	1.282	Q4
10	Archives Of Endocrinology Metabolism	7	2.032	Q4	Endocrine	176	3.925	Q3

### 3.4 Analysis of core author distribution and co-authorship network

**Figure 5:**
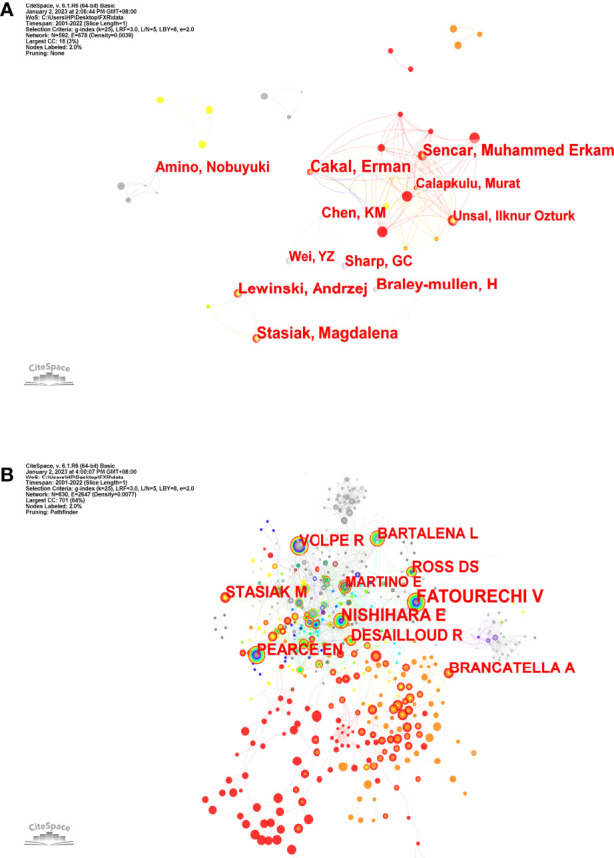
The network maps **(A)** Authors; **(B)** Co-cited authors. The size of node indicates the number of articles published by a certain author, and the links between two nodes mean a collaboration between each other **(A)**. The size of node indicates the co-cited frequency of a certain author, and the links between two circles mean a co-citation relationship between authors **(B)**.

The overall number of authors who contributed to SAT research output was 2,468. **Table 2** lists the authors who are the most productive. The author Braley-Mullen H had the most publications (17), followed by authors Miyauchi A (16) and Sharp GC (16), all of whom had 16 publications. The fourth spot was shared by Cakal E (11), Lewinski A (11), and Stasiak M (11). **Figure 5A** depicts the network visualization map of the authors’ cooperation. Co-cited authors are those who have their work referenced in multiple studies at the same time. **Figure 5B** displays the network visualization map for the co-cited authors. The most often co-cited authors are represented by the largest nodes, including Stasiak M (156), Fatourechi V (127), Nishihara E (124), Brancatella A (96), and Bartalena L (94). The most commonly co-cited authors included two of the top ten most productive authors (Stasiak M, Braley-Mullen H).

**Table 2:** Top 12 productive authors and co-cited authors in SAT research.

Rank	Author	Count	Rank	Co-cited author	Citation
1	Braley-Mullen H	18	1	Stasiak M	156
2	Miyauchi A	16	2	Fatourechi V	127
2	Sharp GC	16	3	Nishihara E	124
4	Cakal E	11	4	Brancatella A	96
5	Lewinski A	11	5	Bartalena L	94
6	Stasiak M	11	6	Volpe R	90
7	Amino N	10	7	Pearce EN	80
7	Chen KM	10	8	Braley-mullen H	74
7	Sencar ME	10	9	Ross DS	70
10	Wei YZ	10	10	Desailloud R	67
10	Fukata S	9	11	Martino E	66
10	Unsal IO	9	12	Bogazzi F	56

### 3.5 Analysis of document co-citation

**Figure 6:**
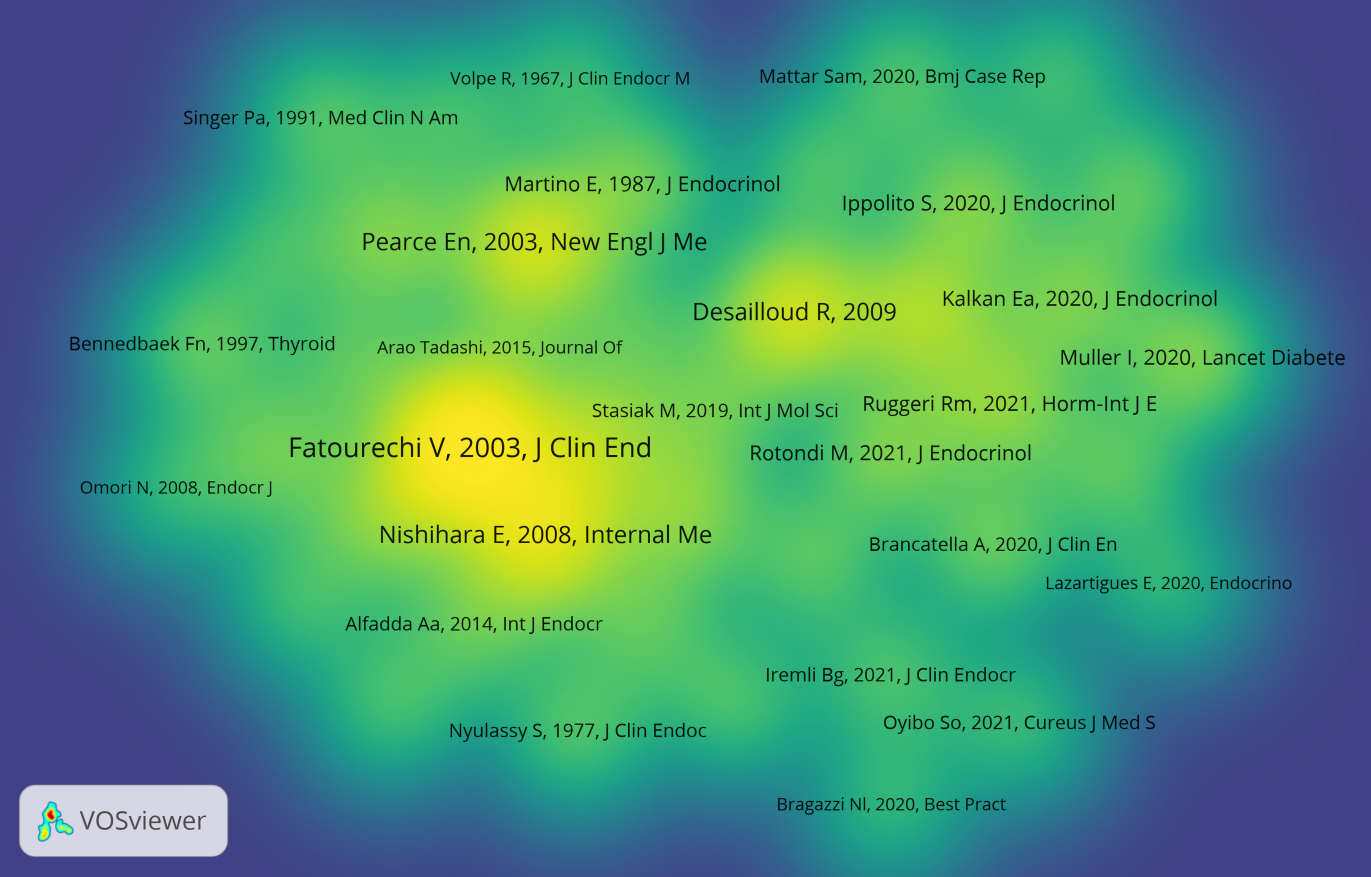
The density map of co-cited documents. The size of the word and round, and the opacity of yellow are positively associated with the co-cited frequency.

Document co-citation is a technique for elucidating co-cited literature by several authors. Precisely, this technique visualizes the co-occurrence of citations in two publications to assess their link (18). Vosviewer examined a total of 568 articles and their 12,163 references that were retrieved from WoSCC during the years of 2001 and 2022 to determine typical homogeneity. **Figure 6** displays a map of co-citation references for SAT research. The findings revealed that the most highly cited reference is a cohort study of clinical features and outcomes of SAT published by *Journal of Clinical Endocrinology & Metabolism* in 2003 (17). This cohort study found that early transient hypothyroidism is common in SAT, and corticosteroid therapy might relieve symptoms but could not prevent early-onset or late-onset thyroid dysfunction. The second-ranked paper was a retrospective clinical study published by *Internal Medicine* in 2008 (19). The study reviewed the medical records of 852 SAT patients from 1996 to 2004, and evaluated the characteristics of SAT at onset, recurrent episodes, and abnormal laboratory findings. The third-ranked paper examined virological data for each form of thyroiditis at various degrees of proof and offered concrete evidence of the existence of viruses or their byproducts in the thyroid gland. However, it was still unknown whether these viruses were the cause of thyroid illness (9). The top 10 co-cited works, which are presented in **Table 3**, have made significant contributions to SAT research and are perhaps the most well-known works in this area.

**Table 3:** The top 10 high co-cited documents in SAT research.

Rank	Title	Journal	Year	Author	Co-citation counts	DOI
1	Clinical features and outcome of subacute thyroiditis in an incidence cohort: Olmsted County, Minnesota, study	Journal of Clinical Endocrinology & Metabolism	2003	Fatourechi V	112	10.1210/jc.2002-021799
2	Clinical characteristics of 852 patients with subacute thyroiditis before treatment	Internal Medicine	2008	Nishihara E	73	10.2169/internalmedicine.47.0740
3	Viruses and thyroiditis: an update	Virology Journal	2009	Desailloud R	66	10.1186/1743-422x-6-5
4	Thyroiditis	New England Journal of Medicine	2003	Pearce EN	66	10.1056/nejmra021194
5	Subacute Thyroiditis After Sars-COV-2 Infection	Journal of Clinical Endocrinology & Metabolism	2020	Brancatella A	50	10.1210/clinem/dgaa276
6	2016 American Thyroid Association Guidelines for Diagnosis and Management of Hyperthyroidism and Other Causes of Thyrotoxicosis	Thyroid	2016	Ross DS	44	10.1089/thy.2016.0229
7	Subacute thyroiditis in a patient infected with SARS-COV-2: an endocrine complication linked to the COVID-19 pandemic	Hormones-International Journal of Endocrinology and Metabolism	2021	Ruggeri RM	40	10.1007/s42000-020-00230-w
8	SARS-CoV-2-related atypical thyroiditis	The Lancet Diabetes & Endocrinology	2020	Muller I	39	10.1016/s2213-8587(20)30266-7
9	Subacute thyroiditis: clinical characteristics and treatment outcome in fifty-six consecutive patients diagnosed between 1999 and 2005	Journal of Endocrinological Investigation	2007	Benbassat CA	37	10.1007/bf03347442
10	SARS-CoV-2: a potential trigger for subacute thyroiditis? Insights from a case report	Journal of Endocrinological Investigation	2020	Ippolito S	37	10.1007/s40618-020-01312-7

### 3.6 Analysis of keyword co-occurrence, clusters, and bursts

**Figure 7:**
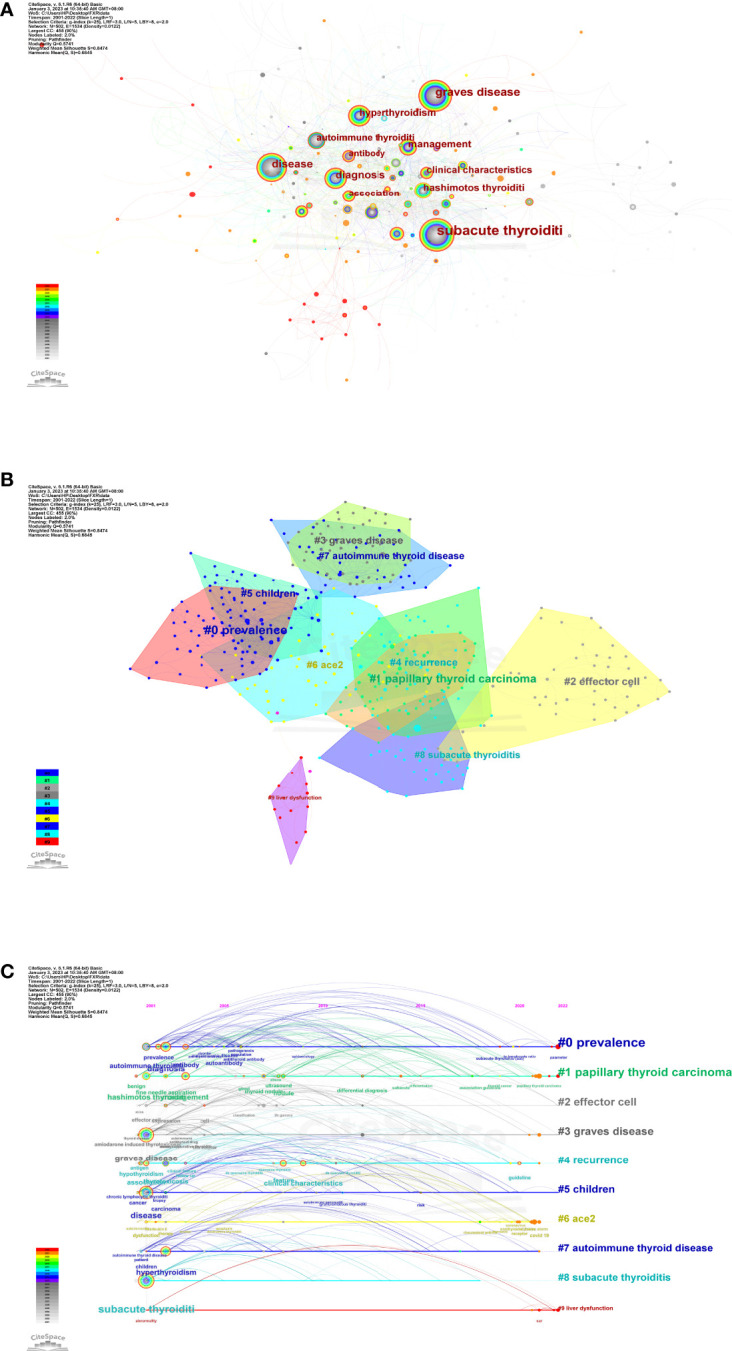
**(A)**The network map of keywords. **(B)** Clustered network of keywords. **(C)** The timeline view of keywords.

**Figure 8:**
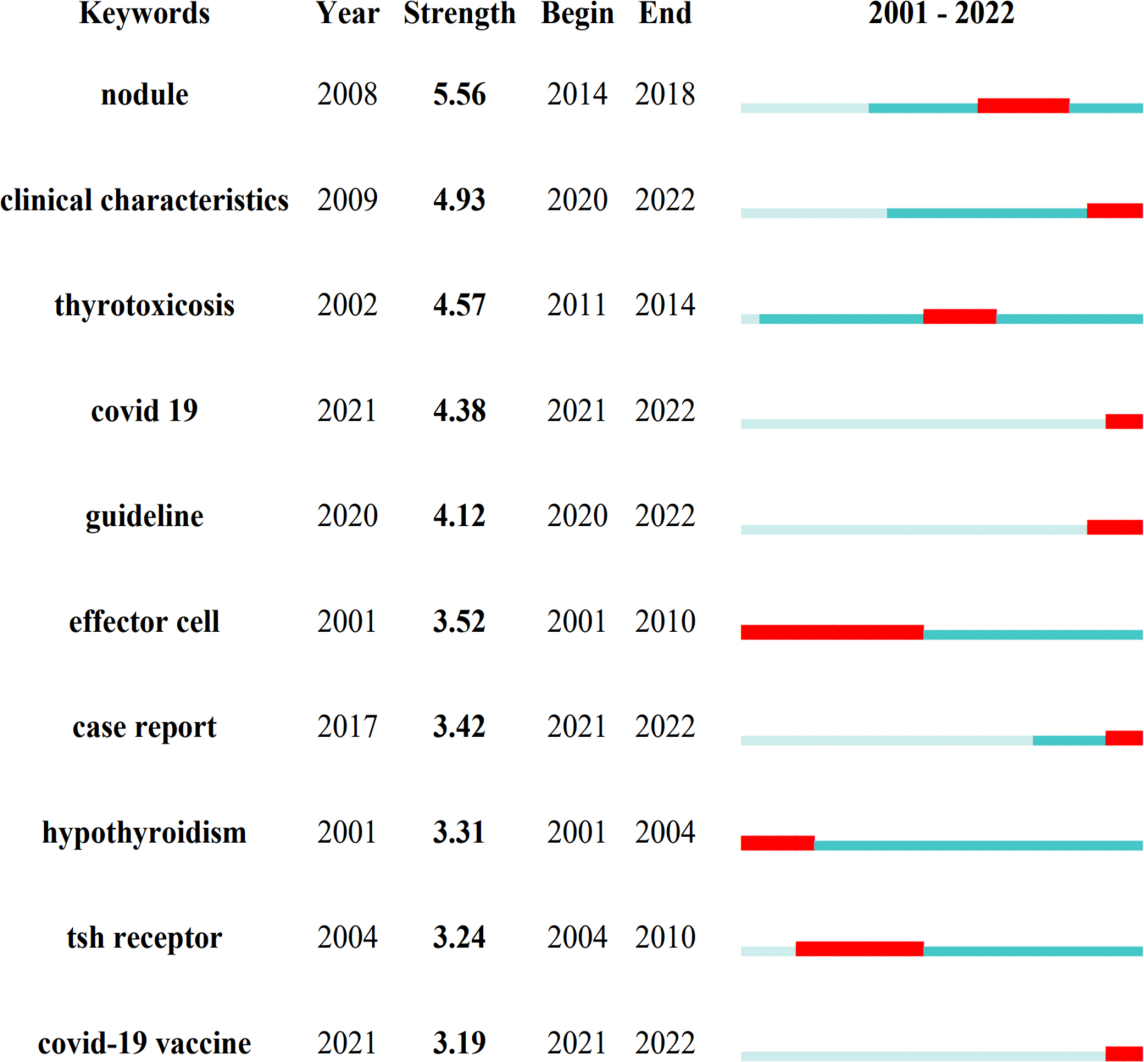
Keywords with the strongest burst strength. The blue line represents the period from 2001 to 2022 and the red line represents the burst's maintenance period.

Keyword co-occurrence and network cluster analysis are both available through CiteSpace. 502 keywords were retrieved in total. **Table 4** and **Figure 7A** show the most frequently occurring keywords, indicating the hotspots of the SAT research. The most relevant terms in the SAT research were identified by the keyword co-occurrence clusters using the hierarchical cluster labeling method, which includes “prevalence” (cluster #0), “papillary thyroid carcinoma” (cluster #1), “effector cell” (cluster #2), “graves disease” (cluster #3), “recurrence” (cluster #4), “children” (cluster #5), “ace2” (cluster #6), “autoimmune thyroid disease” (cluster #7), “subacute thyroiditis” (cluster #8), and “liver dysfunction” (cluster #9) (**Figure 7B**). The amount of cluster labels is inverse to the number of articles each cluster contains. Therefore, cluster #0 has the greatest number of papers. **Supplementary Table S1** contains a list of clusters in summary form. In order to depict the development of high-frequency keywords within each cluster, CiteSpace developed a keywords timeline viewer that could cluster keywords and take time into consideration. The viewer might also make it straightforward to pinpoint the time frame for a specific subject and the development of this research area. Each stage and evolution path of the SAT research’s concentration could be intuitively understood, as shown in **Figure 7C**. We used CiteSpace to find burst keywords to track the hotspots and research boundaries over time. **Figure 8** displays the top 10 keyword bursts from SAT research from 2001 to 2022 that had the most robust strength. The keyword bursts among them that persisted through the end of 2022 included “clinical characteristics” (with a burst strength of 4.93), “covid 19” (with a burst strength of 4.38), “guideline” (with a burst strength of 4.12), “case report” (with a burst strength of 3.42), and covid-19 vaccine (with a burst strength of 3.19), which represented the hot spots in recent years.

**Table 4:** The top 20 keywords associated with SAT research.

Rank	Keyword	Count	Rank	Keyword	Count
1	Subacute Thyroiditi	226	11	Antibody	26
2	Graves Disease	83	12	Thyrotoxicosis	23
3	Disease	66	13	Feature	22
4	Diagnosis	56	14	Nodule	19
5	Management	44	15	Fine Needle Aspiration	17
6	Hyperthyroidism	42	16	Carcinoma	16
7	Hashimotos Thyroiditi	41	17	Autoantibody	16
8	Clinical Characteristics	39	18	Hypothyroidism	16
9	Association	33	19	Prevalence	16
10	Autoimmune Thyroiditi	33	20	Benign	14

## 4 Discussion

### 4.1 General information

It is comparatively challenging to fully grasp the focus of a certain topic, access cutting-edge information, and identify research trends and hot spots in the age of the information boom (20). Bibliometric analysis is often used as a method to solve these problems. As SAT is a self-limiting disease that may resolve spontaneously, there is little research before 2019. However, the COVID-19 outbreak has resulted in a sharp rise in the number of relevant studies, demonstrating that the global scientific community is interested in learning more about the connection between SAT and the COVID-19 infection or vaccination. Since COVID-19 patients’ clinical characteristics of SAT may differ from those of normal SAT patients, and numerous new clinical trends have emerged (21), it is still a promising subject for research and merits financial and human resources investment, which is congruent with the actual clinical situation.

Teamwork and global collaboration in a specific field are made easier with the aid of distribution analysis of countries/regions and institutions. **Figure 3A** demonstrates that the United States and Japan were the two major nations that contributed considerably to SAT research. The United States had the highest centrality (0.36), showing that it was crucial in bridging international cooperation (16), but it is a pity that there was not much cooperation among other countries/regions. Additionally, the fact that three of the top five universities are from the United States shows that American academics have dominated SAT research for the past 20 years and have the greatest influence. This distribution has to do with academic funding and economic growth. At the same time, most institutional cooperation is domestic scientific cooperation, and inter-national institutional cooperation is insufficient (**Figure 3B**). Given the above, it is essential to strengthen international relations and institutional collaboration to encourage the ongoing growth of this field and help more SAT sufferers.

An analysis of journals and co-citations of journals can demonstrate their contribution to the field, and researchers may use these results to choose appropriate journals for manuscripts relating to SAT. The journal *Thyroid* (n = 36) published the most papers on this topic. *Thyroid*, an official journal of the American Thyroid Association, is the top journal in the field, whose publications cover thyroid diseases from cellular molecular biology to clinical management. Among the top 10 most co-cited journals, *Journal of Clinical Endocrinology & Metabolism* (n = 1350) possessed the most co-citations. It is a journal associated with research on the clinical practice of endocrinology and metabolism. Five of the top 10 co-cited journals, including *Lancet* (IF = 202.731) and *New England Journal of Medicine* (IF = 176.079), are in the Q1 JCR division, proving that some high-caliber and high-impact journals values SAT research.

Among the authors who contributed to the research of SAT from 2001-2022, Braley-Mullen H. from the University of Missouri System published 18 articles in this field. Prof. Braley-Mullen H. was an expert in the field of thyroid diseases and was committed to the molecular mechanism research of various types of thyroiditis. Stasiak M. from Polish Mother’s Memorial Hospital Research Institution was the most co-cited author in this field. Prof. Stasiak M. was engaged in clinical research on thyroid diseases and conducted in-depth research on genetic susceptibility for SAT.

Among the top 10 high co-cited documents in SAT research, 5 were published before 2010 (4, 9, 17, 19, 22), which mainly focused on the clinical characteristics and pathogenesis of SAT, while 4 articles published after 2019 were studies on SAT in relation to the COVID-19 pandemic (23–26). The number of co-citations reached the top 10 within 2 years, reflecting that the research of SAT under the COVID-19 pandemic is a current research hotspot. However, the absence of basic research among the top 10 high co-cited articles suggests that basic research on the SAT is not a trend for the next few years.

The clustered network and timeline view of keywords display the evolution of high-frequency keywords and show the research progression path evolution in the research of SAT **(** **Figure 7** **)**. Three research core themes can be distilled into the following characteristics using these analyses: 1. Prevalence (cluster #0, cluster #5 and cluster #6); 2. Diagnosis (cluster #1, cluster #3, and cluster #7); 3. Treatment (cluster #2, cluster #4, and cluster #9). The keyword bursts are thought to be signs of modern subjects or developing trends **(** **Figure 8** **)**. The keyword bursts among them that lasted until the end of 2022 included “covid 19”, “covid-19 vaccine”, “clinical characteristics”, “case report”, and “guideline”, which represented the hot spots in recent years. The clinical characteristics of SAT have been changing significantly in recent years, and findings are usually initially presented in the form of case reports. COVID-19 and COVID-19 vaccination can be potent SAT-triggering factors, and the clinical course of SAT in patients affected by them is different from a typical one. It is imperative to explore new guidelines for the diagnosis and treatment of SAT. Stasiak M. et al. have proposed new diagnostic criteria for SAT that complement new aspects related to the COVID-19 pandemic and may help improve the effectiveness of diagnosis and treatment of the disease (21).

### 4.2 Research core themes

#### 4.2.1 Prevalence

Middle-aged women had the highest SAT incidence rate, accounting for 75% to 80% of all SAT patients (3). It is worth noting that there have been recent reports of SAT in kids, despite the fact that SAT in children is thought to be incredibly rare (27, 28). Clinicians should be aware that SAT may exist in children.

As a result of the COVID-19 outbreak, up to 10% to 20% of COVID-19 patients who are hospitalized also have symptoms of SAT (25, 29). However, the impact of COVID-19 on the prevalence of SAT is still up for discussion. The results of a retrospective single-center study conducted in Turkey from 2018 to 2020 found increases in seasonal variation and an increase in the number of men who had SAT but no changes in the prevalence or clinical course of the illness (30). On the contrary, a large cross-sectional study conducted in South Korea revealed that the incidence of SAT was much greater in 2020 than it was from 2017 to 2019, and corticosteroids were prescribed more frequently, but the peak age and sex ratio of onset were no different from previous years (31). The generalization of incidence rate statistics is constrained by the current study’s single-center, retrospective design and potential cross-national and regional variances. It is essential to perform a multicenter study based on the general population to evaluate the existing results.

#### 4.2.2 Diagnosis

The symptoms and signs of SAT are not typical. Despite advancements in diagnostic methods, new changes in the clinical presentation make the diagnosis much more challenging and more likely to result in a false negative. Patients frequently see multiple doctors before receiving the diagnosis of SAT, and the time it takes can vary from two weeks to six months (32). In a retrospective study, an upper respiratory tract infection was the initial diagnosis for one-third of SAT patients (33). Misdiagnosis of infection leads to overuse of antibiotics, with fever, elevated C-reactive protein (CRP) levels, and white blood cell (WBC) being the most common features in patients treated with antibiotics.

False negative SAT diagnoses cause therapy delays and poor quality of patient’s life, but they do not pose a life-threatening risk. However, the false positive diagnosis of thyroid primary and metastatic malignancies as SAT will delay treatment and endanger patients’ life (34). It was thought to be extremely rare for SAT and thyroid carcinoma to coexist, and such cases were typically reported as case reports (35, 36). Following up with 710 SAT patients for a long time demonstrated that initial ultrasound screening for thyroid nodules had a sensitivity of 72.4%, specificity of 89.0%, positive predictive value of 80.4%, and negative predictive value of 83.8% in SAT patients (37). In 3.1% of individuals with SAT, thyroid papillary carcinoma (PTC) was found, and up to 30% of PTC instances go unreported at the initial scan and are only found at a subsequent ultrasound (37). Therefore, ultrasound retesting should always be performed after SAT-related thyroid lesions have subsided. Fine needle aspiration biopsy (FNAB) tests should be carried out to rule out malignancy if the ultrasonography results are questionable (34).

#### 4.2.3 Treatment

Pain relief and inflammation management are the primary goals of SAT therapy. NSAIDs and steroids have long been recommended for the treatment of SAT. Observational findings suggested that NSAIDs were less effective in SAT treatment than steroids, which were considered protective factors in reducing recurrence (6, 38, 39). Recurrence of SAT and steroid dependence remain essential issues in the treatment of SAT. The danger of recurrence from reducing glucocorticoid doses and the risk of consequences from glucocorticoid dependence must be balanced carefully. Evidence shows that a higher prevalence of hypothyroidism is linked to large cumulative dosages of prednisolone (40).

The optimal steroid treatment for SAT is still controversial. A high risk of recurrence is known to be linked to a too fast tapering of steroid dose. A randomized controlled trial, however, has revealed that short-term prednisone therapy is comparable to long-term efficacy and has a better safety profile (39). Additionally, a cohort study discovered that SAT recovery might be possible with low-dose steroid therapy (41). The results of these related studies should warrant further in-depth clinical trials involving more patients to assess how to avoid long-term steroid therapy.

In addition, it has been reported that ultrasound-guided intrathyroid administration of corticosteroids can significantly reduce the duration of SAT therapy compared to oral administration (42, 43), but further evidence-based medical evidence is needed. For SAT patients who have relapsed and are resistant to prednisolone, colchicine has been reported to have a potential therapeutic benefit (44). Nevertheless, solid proof will require a large, double-blind, controlled, prospective multicenter trial, and therefore it needs to be used with caution.

### 4.3 Research hotpots

#### 4.3.1 Clinical characteristic

The clinical characteristics of the disease have seen various alterations in recent years. The frequency of painless SAT has increased, reaching 6.25% (3), as more and more cases have been documented (45, 46). Fever was also observed to occur less frequently than previously believed and was commonly associated with microhematuria (3). It was once believed that the absence of thyroid antibodies constituted a distinctive feature of the SAT. However, elevated levels of anti-thyroid antibodies, such as thyroid peroxidase antibodies (aTPO), thyroglobulin antibodies (aTG), and even thyrotropin receptor antibodies (TRAb) are more often present (3, 47).

A typical late SAT symptom is persistent hypothyroidism. According to earlier research, the extent of inflammation and thyroid hormone levels in SAT patients may be reflected by ultrasound, but it is challenging to forecast permanent hypothyroidism (48). However, a recent study indicated that the probability of chronic hypothyroidism is connected to the decrease in thyroid volume shown by ultrasound within one month of beginning (49). Thyroid-stimulating hormone (TSH) and CRP levels were revealed to be risk factors for hypothyroidism in SAT patients, particularly in those with TSH levels less than 0.10 mIU/L and CRP levels greater than 97.80 mg/L (50).

The relationship between SAT susceptibility and specific HLA categories has been discovered (21). In 70% of SAT patients, HLA-B*35 was detected (51), but other genotypes were also discovered to be connected to the genetics of SAT. Along with the connection to HLA-B*35 that has already been discussed, SAT is also linked to the presence of HLA-B*18:01, HLA-DRB1*01, and HLA-C*04:01 (52). Recent research has demonstrated that the risk of SAT recurrence is HLA-dependent, with the presence of both HLA-B*18:01 and HLA-B*35 serving as the decisive factor (7), revealing that SAT recurrence may be genetically related. Additionally, it was discovered that the sonographic pattern of the SAT was related to HLA (53). Multiple hypoechoic hazy lesions, which are common, were infrequently detected in HLA-B*18:01 positive patients. Most of the patients with HLA-B*18:01 alone had a unilateral, homogenously hypoechoic single SAT region that filled the entire affected lobe and resembled a large thyroid nodule (53). The form of the SAT lesions, which were spotty or spherical, imitating true thyroid nodules, was the main departure from the expected pattern in patients with co-presence of HLA-B*18:01 and HLA-B*35 (53).

#### 4.3.2 Influence of COVID-19

In May 2020, the first SARS-CoV-2 infection-related SAT case was reported (23). The original report’s two-week window between PCR positive and SAT incidence rate was typically accepted. However, SAT caused by SARS-CoV-2 may diverge dramatically from the classic one. The majority of the stages are painless, but tachyarrhythmias and worsening of a general condition are frequently regarded as the main symptoms, especially in patients hospitalized with COVID-19 (21, 25). In certain patients, SAT usually occurs a few weeks after COVID-19 (21, 24), although in other cases it may take a few days for SAT to occur following the commencement of COVID-19 (26), and the two conditions may even manifest at the same time (21, 25, 29). This phenomenon can be HLA-dependent, and the presence of homozygosity at HLA-B*35 may be a potential major contributor to the early onset of SAT symptoms (54). Clinicians need to be aware that COVID-19 infection may result in thyroid dysfunction. Early detection and prompt anti-inflammatory therapy contribute to successful treatment.

Previous viral infection is thought to be the trigger for the SAT. Significant histotropism is seen by SARS-CoV-2, including a strong affinity for thyroid tissue. Angiotensin-converting enzyme 2 (ACE2), a possible receptor that allows the virus to enter cells, is a significant component of new coronavirus infection, and thyroid cells are abundant in ACE2 (55, 56). A study has demonstrated that thyroid follicular cells exhibit significant levels of the mRNA encoding ACE2 receptor, making them a possible entry point for COVID-19 (57). Many SAT cases directly associated with SARS-CoV-2 infection have been described (23, 58–61). Although the scale and quality of the published COVID-19 related SAT data are insufficient, in view of the development of the epidemic, we should still consider that SARS-COV-2 is the most important trigger factor for SAT at present, and its relevant mechanism needs further study.

Finding a therapeutic vaccination that is both effective and safe has become a top priority as COVID-19 spreads to become a pandemic. The vaccine contains adjuvants, which cannot avoid adverse reactions and can cause autoimmune/inflammatory syndrome induced by adjuvants (ASIA) (62). SARS-CoV-2 vaccination can lead to subacute thyroiditis as a phenomenon of ASIA syndrome. The first report of SAT as a phenomenon of ASIA syndrome after inactivated COVID-19 vaccination was reported in August 2021 (63). These cases shared diagnostic characteristics and clinical courses with the classic form of SAT. The mechanism of thyroid dysfunction caused by COVID-19 vaccines has yet to be clearly elucidated at present. Possible mechanisms are considered to include molecular mimicry caused by exposure to abnormal reactivity of adjuvants and/or viral proteins. Adjuvants are substances that promote the immunogenicity of vaccines, and SAT is assumed to be brought on by adjuvant-dependent autoimmune inflammatory alterations. However, it is becoming evident that the cause of SAT may be more complex than just the adjuvant as more cases of SAT associated with unadjuvanted COVID-19 vaccinations are reported (64, 65). The immune response to mRNA and whole viral vaccinations frequently uses spike protein as a stimulant. By binding to HLA-B*35 molecules in macrophages and activating cytotoxic T cells, spike proteins can cause SAT in susceptible individuals (66). Since spike protein-binding ACE2 receptors are abundant in thyroid follicular cells, their activation may be why thyroid follicular cells are being destroyed.

SAT occurrence after COVID-19 vaccination was also HLA-dependent and associated with a specific HLA profile covering the simultaneous presence of HLA-B*35:03 and HLA-C*04:01 (67, 68). Thyrotoxicosis and a more intense inflammatory response were related to homozygosity for HLA-B*35 and HLA-C*04 (67). According to a recent study, which is the first to suggest that the frequency of the HLA-A*11 allele is associated with SAT, it was found that SAT caused by the SARS-CoV-2 vaccine had a higher frequency of the HLA-A*11 allele and the A*11-B*35-C*04 haplotype than in the group unrelated to the SARS-CoV-2 vaccine (69). These findings suggest that HLA-related susceptibility may play a significant role in the development of SAT after COVID-19 vaccination, and the results need to be confirmed in a larger patient population with complete HLA genotyping results available.

Most SAT patients caused by vaccination have a mild clinical course that improves with the use of NSAIDs or steroids (70). It was recommended that patients be treated with NSAIDs in order to obtain adequate vaccine antibody response (66). A systematic review has shown that thyroid diseases may occur within 2 months after COVID-19 vaccination, and SAT is the most common of all thyroid diseases (71). Revaccination in COVID-19 vaccine-induced SAT cases currently appears to be safe (72), but the quantity and quality of published data on thyroid discomfort following COVID-19 vaccination are limited, and further evidence is needed on COVID-19 vaccine-induced SAT.

### 4.4 Strengths and limitations

According to our knowledge, this study is the first bibliometric analysis of SAT research to offer researchers guidance. Compared to a typical review, an analysis based on bibliometric tools, such CiteSpace and VOSviewer, offers a better depiction of changing research trends and hotspots and a relatively thorough and objective data analysis. However, this study has some limitations. First, despite recent increases in the number of publications published in SAT research, the aggregate total is still rather low. Second, owing to CiteSpace’s format constraints, we only counted publications in the WoSCC database, which may have disregarded papers only in other databases such as PubMed, Medline, and Scopus. Due to the extensive cross-replication of records in other databases and the specialized authority of the WoSCC database, this study may still be utilized to illustrate the general situation and overall trend in this field. Third, non-English publications were not included in the study since English was the most often used language, which might have influenced the results due to source bias.

## 5 Conclusion

We conducted the first bibliometric analysis utilizing tools like CiteSpace and VOSviewer to examine the trends and hotspots in SAT research. Due to the COVID-19 pandemic, there has been a sharp rise in the number of publications in the SAT field in recent years, indicating a growing interest among researchers in the field. The clinical characteristics and the genetic background of SAT under the influence of COVID-19 are currently research hotspots. Our study clarified the fundamental scientific understanding of SAT and offered crucial hints for emerging research trends and hotspots. We hope that this study will aid researchers in better grasping the general trend in this field and offer guidance for future research.

## Data availability statement

The original contributions presented in the study are included in the article/**Supplementary Material**. Further inquiries can be directed to the corresponding author.

## Author contributions

CX conceived and designed the study. RJ and J-yL contributed to data collection. CX, RJ, and J-yL conducted the data analysis and interpretation. CX drafted the initial manuscript. CX revised the manuscript. All authors contributed to the article and approved the submitted version.
