# Supplementary material for: Emerging trends and hot spots in subacute thyroiditis research from 2001 to 2022: A bibliometric analysis
Source: Front Endocrinol (Lausanne). 2023 Mar 17;14:1144465. doi: 10.3389/fendo.2023.1144465 (PMC10064097; doi:10.3389/fendo.2023.1144465)
Supplement: Supplementary file 2 [file Table_1.docx]

**Supplementary Table 1.** Summary of the top 10 largest clusters of citing articles on the research of SAT (Silhouette value >0.7 means the clustering results are reliable (1)).

| **Cluster ID** | **Cluster Name** | **Size** | **Silhouette** | **Top Terms** |
| --- | --- | --- | --- | --- |
| 0 | prevalence | 64 | 0.871 | prevalence; population; epidemiology; autoantibody; autoimmune disease |
| 1 | papillary thyroid carcinoma | 54 | 0.859 | papillary thyroid carcinoma; thyroid nodule; fine-needle aspiration; diffuse thyroid disease; contrast-enhanced ultrasound |
| 2 | effector cell | 47 | 0.931 | effector cell; fas mediated apoptosis; cd8(+) t cell ; expression; flip |
| 3 | graves disease | 46 | 0.82 | graves disease; antithyroid drug; subacute thyroiditis; subacute thyroiditi; term follow up |
| 4 | recurrence | 43 | 0.822 | recurrence; hypothyroidism; subacute thyroiditis; tsh; de quervain thyroiditis |
| 5 | children | 43 | 0.862 | children; dementia; thyroid nodules; aspiration biopsy; retroperitoneal fibrosis |
| 6 | ace2 | 41 | 0.752 | ace2; amiodarone-induced thyrotoxicosis; anti-thyroid drugs; thyroid hormones; destructive thyroiditis |
| 7 | autoimmune thyroid disease | 40 | 0.836 | autoimmune thyroid disease; patient; amyloid goite; autoimmune disorder; children |
| 8 | subacute thyroiditis | 39 | 0.732 | subacute thyroiditis; covid-19; thyroid hormone; extracellular matrix; computed tomography |
| 9 | liver dysfunction | 13 | 0.965 | liver dysfunction; coronavirus disease 2019; vaccination; asia syndrome; neck pain |

1. Chen C, Dubin R, Kim MC. Emerging trends and new developments in regenerative medicine: a scientometric update (2000 - 2014). Expert Opin Biol Ther. 2014;14(9):1295-317.
